# The Encyclopedia of Chemistry, Practical and Theoretical; Embracing Its Applications to the Arts, Metallurgy, Mineralogy, Geology and Pharmacy

**Published:** 1850-05

**Authors:** 


*The Encyclopedia of Chemistry, Practical and Theoretical; embracing its applications to the Arts, Metallurgy, Mineralogy, Geology and Pharmacy.* By James C. Booth, A. M., M. A. P. S., &c. Assisted by Campbell Morfit. 8vo. pp. 974. Philadelphia: Henry C. Baird, successor to E. L. Carey, 1850.

The Encyclopedia embraces too wide a scope, as indicated by the above title, to be included in 974 large octavo pages of small print. It, of course, is not to be expected that the whole subject can be here produced in any great detail, and hence this is not aimed at, except in the applications to manufactures and metallurgy. In these departments, the authors have extended their observations, and given various modifications of the processes employed, and under the heads of the articles themselves, references to their use, with the particular parts they are expected to perform in these processes, and such incidental information as may be practically useful to the manipulator. Thus acetic acid, alcohol, alcoholometry, alum, beer, bleaching, calico printing, caoutchouc, &c., have a larger proportionate space allotted to them than substances which, belonging to the other departments, have no particular applications in the arts or metallurgy. In thus giving particular attention to distinct branches of the science, the more elementary parts have not been overlooked, but, as the foundation of all the rest, have devoted to them such notice as is requisite to their proper applications and correct appreciation. In thus directing their aims in a special direction, it does not, however, appear that space is obtained by omission of any subjects as too unimportant in character, but rather by apportioning the amount on each topic to its relative value, and treating these subjects in as concise a manner as may be consistent with perspicuity. In the dictionary form necessary to a work of this kind, considerable effort is necessary to prevent a repetition of statements and facts under the different heads in which they may be appropriately introduced, and at the same time to avoid too frequent references from one head to another. This effort has apparently been made with considerable success, by passing in review all the parts connected with any particular subject, referring to their distinct heads, and giving but few words to that which has been elsewhere noticed, and where a sufficient explanation has been given under the general subjects, referring to the principal subjects under each distinctive title. Thus, under bile and blood, the constituents of these complex organic fluids are given, and either separately commented on, or reference made to them, as "see Hematin and Globulin;" or under the special names we have a reference to these subjects, as "Fulvic Acid," and "Taurin, see Bile," &c.

Some few omissions and inadvertencies of expression, however, have been noticed, which have escaped the attention of the authors; thus, under "Hydrometer," no mention is made of the applications of the same principle in the urinometer. Under Urea, it is stated that it is prepared artificially by mixing " 28 *parts* of dry ferrocyanide of potassium, and 14 of peroxide of manganese," and " 20 *parts* of crystallized sulphate of ammonia;" and immediately below " from 5—6 *ozs.* of urea may be obtained from the above *proportions.*"

In revising the proofs, uniformity of orthography has not received as full attention as the scientific portions of the work; thus the phrase " wear and *tare* " is twice repeated within three pages. In the one page there is fellinic and twice fellanic acid; although not here important, the change of a single letter may in other instances of organic compounds indicate a marked difference in composition, of which an example may be given in butyrene $C_8 H_8$, butyrine $C_{14} H_{14} O$, butyrone $C_{14} H_{14} O_2$.

The illustrations consist of wood cuts introduced under the different subjects requiring them, and nine plates of more general reference, and not well adapted to the body of the work.

In conclusion, we would state, that as a work of reference, the Cyclopedia will be found very useful, as embodying a large amount of practical information gleaned from a great variety of sources, and from personal experience up to the time of its publication, upon the accuracy of which the well known knowledge and judgment of the authors would lead us to place great reliance.

